# 

**Published:** 1902-06-28

**Authors:** 


### NOTICE TO CORRESPONDENTS.

All MSS., Letters, Books for Review, and other matters intended for the Editor should be addressed The Editor, "The Hospital," The Hospital Building, 28 & 29 Southampton Street, Strand, W.C.

The Editor cannot undertake to return rejected MS., even when accompanied by stamped directed envelope.

**Notice to Advertisers and Subscribers.**

All Advertisements, Orders for Copies of The Hospital, and Business Communications should be addressed to **THE MANAGER, (Not to the Editor)**, The Hospital, 28 & 29 Southampton Street, Strand, London, W.C.

The Cost of Subscription, post free, is as follows.—Per week, 2½d.; per quarter, 2s. 8d.; per half-year, 5s. 3d.; per annum, 10s. 6d. Foreign:—Per annum, 15s. 2d., payable, in all cases, in advance.

**NOTICE.—Vol. XXXI. of "The Hospital" (October 1901, to March 1902), in handsome cloth binding is now on sale at the office of this Journal, price, 7s. 6d. Cases for binding the Numbers contained in this Volume 1s. 6d. Index to Vol. XXXI., 2½d., post free.**

**The Monthly Part for July will be ready on July 1. Price 6d., by post 8d.**

### THE STUDENT OF MEDICINE.

### APPOINTMENTS.

W. H. Andrews, L.R.C.P., L.R.C.S.Edin., appointed Certifying Factory Surgeon for the Modbury District of Devon. A. S. Bruzaud, L.R.C.P.Lond., M.R.C.S.Eng., appointed District Medical Officer of the Saddleworth Union. William Calwell, M.D.R.U.I., of Belfast, appointed Honorary Consulting Physician to the County Antrim Infirmary, Lisburn. F. W. J. Coaker, F.R.C.S.Eng., L.R.C.P., appointed Medical Officer of Health to the Bromsgrove Rural District Council, and Medical Officer and Public Vaccinator for the Bromsgrove District of the Bromsgrove Union. W. B. Cockill, L.R.C.P.Lond., M.R.C.S.Eng., appointed Certifying Factory Surgeon for the Kendal District of Westmorland. **Middleton Connon**, M.B., C.M., D.P.H., appointed Visiting Surgeon to the Montrose Royal Infirmary. **W. H. Cooper**, M.R.C.S., L.R.C.P.Lond., appointed Certifying Factory Surgeon for the Staveley District of Westmorland. **R. W. Cruikshank**, M.D., C.M.Aberd., appointed Certifying Factory Surgeon for the Eynsham District of Oxford. **Louis Demetriadi**, F.R.C.S.E., L.R.C.P.I., D.P.H., appointed Visiting Surgeon to the Children's Homes, Outlane, under Huddersfield Board of Guardians. **Quinto Ercole**, M.D., Ch.D.Bologna, appointed Government Medical Officer and Vaccinator at White Cliffs, New South Wales. **C. C. Easterbrook**, M.A., M.D., M.R.C.P.Edin., appointed Medical Superintendent to the Ayr District Asylum. **G. B. Forge**, M.R.C.S., L.R.C.P.Lond., appointed Certifying Factory Surgeon for the Rochford District of Essex. **Richard H. O. Garbutt**, L.R.C.P., L.R.C.S.Edin., appointed Certifying Factory Surgeon for the Tow Law and Stanhope Urban Districts and the Weardale Rural District. **Innes Griffin**, M.R.C.S.Eng., L.S.A., appointed Certifying Factory Surgeon for the Banbury District of Oxford. **E. J. F. Hardenberg**, M.R.C.S Eng., L.R.C.P.Lond., appointed Senior Resident Medical Officer to the North West London Hospital. **E. Hulbert**, M.D.Durh., appointed Resident Medical Officer to the Marylebone General Dispensary. **R. A. Lambert**, M.D.Edin., L.R.C.S., appointed Certifying Factory Surgeon for the East Ilsley District of Berks. **John T. Leon**, M.D., B.Sc.Lond., appointed Honorary Assistant Physician to the Royal Portsmouth and Gosport Hospital. **William A. McCutchan**, L.R.C.P. and S.Edin., appointed Assistant Medical Officer to the Cambridgeshire Asylum. **Ernest Mackenzie**, M.D.Glasg., C.M., appointed Certifying Factory Surgeon for the Cheadle District of Staffs. **Donald McLean**, M.B., appointed Medical Officer of Health for the Shire of Dunmunkle, East Riding, and Public Vaccinator for the North-Western of Victoria. **J. G. McNaughton**, M.D., M.R.C.S.Edin., appointed Pathologist to the Clinical Hospital, Manchester. **Herbert C. Male**, M.D., M.R.C.S., appointed an Honorary Medical Officer to the Croydon General Hospital. **G. W. Pickering**, L.R.C.P., L.R.C.S.Edin., appointed Certifying Factory Surgeon for the Haltwhistle District of Northumberland. **William Reginald Stowe**, M.R.C.S.Eng., L.R.C.P.Lond., appointed Public Vaccinator for the District of Palmerston North, New Zealand. **A. E. Street**, M.R.C.S., L.R.C.P.Lond., appointed District Medical Officer of the Ampthill Union. **E. F. Vaughan**, M.R.C.S., L.R.C.P.L., appointed Assistant Medical Officer to the St. Marylebone Infirmary, Notting Hill. **R. J. Waugh**, M.R.C.S.Eng., L.R.C.P.Lond., appointed Junior Resident Medical Officer to the North-West London Hospital. **Rayner Winterbotham**, M.R.C.S., L.R.C.P.Lond., appointed Certifying Factory Surgeon for the Brixworth District of Northampton. **Cecil Woodhouse**, B.A.Cantab., M.D., appointed Medical Officer and Public Vaccinator for the Esher District of the Kingston Union.

**"The Hospital" Institutional Library.**

"Hospitals and Asylums of the World." 4 Vols., with a Portfolio of Plans, complete £12 12s.

"Burdett's Hospitals and Charities, 1902." 5s.

"Cottage Hospitals, General, Fever, and Convalescent." 10s. 6d.

"Hospital Expenditure: The Commissariat." 2s. 6d.

"A Legal Handbook for the Use of Hospital Authorities." 2s. 6d.

"The Cottage Hospital Case Book or Register of Patients." 10s. and 12s. 6d.

"The Furnishing and Appliances of a Cottage Hospital." 6d.

All these are published by the Scientific Press, Ltd., and may be obtained through any bookseller, or direct from the publishers, 28 and 29 Southampton Street, Strand, London, W.C.

### An ATLAS of BACTERIOLOGY.

Containing 111 Original Photo-Micrographs, with Explanatory Text. By CHARLES SLATER, M.A., M.B., M.R.C.S. (Eng.), F.C.S., and EDMUND J. SPITTA, L.R.C.P. (Lond.), M.R.C.S. (Eng.). Demy 8vo., cloth gilt, 7s. 6d. net.

"It is impossible to deny that this work fills a blank in the life of the student of bacteriology. The photographs are excellent and the letterpress, linking together and explaining the teaching of the illustrations, is clear, concise, and accurate."—*Nature.*

London: THE SCIENTIFIC PRESS, Ltd., 28 & 29 SOUTHAMPTON STREET, STRAND, W.C.

